# Taking advantage of quasi-periodic signals for S2S operational forecast from a perspective of deep learning

**DOI:** 10.1038/s41598-023-31394-1

**Published:** 2023-03-13

**Authors:** Yang Zhou, Qifan Zhao

**Affiliations:** 1 Collaborative Innovation Center on Forecast and Evaluation of Meteorological Disasters, Key Laboratory of Meteorological Disaster, Ministry of Education, Nanjing University of Information Science and Technology, Nanjing, China; 2 School of Atmospheric Sciences, Nanjing University of Information Science and Technology, No. 219 Ningliu Road, Pukou District, Nanjing, 210044 Jiangsu China

**Keywords:** Atmospheric dynamics, Statistics, Computer science

## Abstract

The quasi-periodic signals in the earth system could be the predictability source for sub-seasonal to seasonal (S2S) climate prediction because of the connections among the lead-lag time of those signals. The Madden–Julian Oscillation (MJO) is a typical quasi-periodic signal, which is the dominant S2S variability in the tropics. Besides, significantly periodic features in terms of both intensity and location are identified in 10–40 days for the concurrent variation of the subtropical and polar jet streams over Asia in this study. So far, those signals contribute less and are not fully applied to the S2S prediction. The deep learning (DL) approach, especially the long-short term memory (LSTM) networks, has the ability to take advantage of the information at the previous time to improve the prediction after then. This study presents the application of the DL in the postprocessing of S2S prediction using quasi-periodic signals predicted by the operational model to improve the prediction of minimum 2-m air temperature over Asia. With the help of deep learning, it finds the best weights for the ensemble predictions, and the quasi-periodic signals in the atmosphere can further benefit the S2S operational prediction.

The numerical climate prediction on sub-seasonal to seasonal (S2S) time scales has been operational in weather/climate forecast centers worldwide for years^1^. Compared to the short-range weather forecast, its extended range of lead time can provide valuable hours for thoughtful decisions and adequate preparations, but skillful S2S prediction with a long lead time is a remaining challenge for meteorologists^2,3^. As an important criterion to assess S2S operational models, a lot of effort has been invested in the improvement of predicting quasi-periodic signals such as the Madden–Julian Oscillation (MJO), which is the dominant S2S signal in the tropical atmosphere^4–6^. In the operational models, the lead time of skillful MJO prediction can be over 30 days^7,8^, which is much longer than those for the other atmospheric variables in extratropical areas such as 2-m air temperature^9^. Though MJO significantly influences the S2S prediction, the improvement of MJO prediction brings limited benefit for S2S prediction about the other atmospheric variables^9–11^. This is partly because the numerical model may not well express the complex interactions between MJO and the atmosphere. Moreover, the effects of MJO on S2S prediction are not extensively revealed and mainly derived from traditional statistical methods, which are generally restricted to linear approaches. While numerical models are developed for improving S2S prediction, how can we seek a way to take advantage of the quasi-periodic signals (e.g., MJO) in S2S prediction?

Nowadays the major tool for S2S prediction (between two weeks and a season) is the dynamic model, which is driven by both initial and boundary conditions. Forecasts of less than two weeks depend on initial conditions^12^, and the slow-evolving aspects incorporated in the boundary conditions are dominant factors for climate predictions longer than a season^13^. For the S2S prediction, the model losses most of the memory of its initial state, and the time period is too short for the model atmosphere to acquire notable responses to the slow-evolving parts of the boundary conditions. Thus, the variabilities in the atmosphere on S2S time scales become an important source of predictability^14–16^. For example, MJO impacts the evolution of midlatitude circulation several weeks later^17–19^, and significant improvement in MJO prediction is achieved in the S2S operational models^7,8^. The Beijing Climate Center (BCC) model, which is the S2S operational model of the Chinese Meteorology Administration (CMA) joining the S2S project (http://www.s2sprediction.net), can provide skillful MJO prediction about 20 days ahead^20,21^, but the other variables such as 2-m air temperature in the model still have the short lead time. Besides prediction itself, S2S climate prediction is an issue with the postprocessing of massive model outputs^1^. The major task of postprocessing is to achieve skillful S2S prediction, which can adopt deep learning (DL)^22^. DL is used to postprocess outputs from ensemble members of S2S forecasts to obtain better predictions of MJO and atmospheric variables than those of the direct ensemble mean^23,24^.

Confronting massive atmospheric datasets and complex nonlinear problems, efficient statistical tools are required beyond linear approaches to express the nonlinearity and complement current understanding. Nowadays state-of-the-art numerical models help understand and predict the climate system much better than before, but complex phenomena and useful information still need to be dug in numerous datasets yielded year on year^25^. Although the models are based on nonlinear dynamics, analysis methods for the model outputs are mostly linear statistics^26,27^, which to a certain extent preclude most of the nonlinearity. According to the outstanding performance in image processing and language recognition, DL becomes a powerful engine for massive data analysis with nonlinearity being considered^28,29^. Machine learning (ML) and DL have become modern tools in earth science with large amounts of observation and model data^30–32^. Due to the success in dealing with images, DL is applied in the identification of weather patterns^33,34^, the category of precipitation types^35^, and the classification of the wave breaking in the ocean^36^. Based on satellite and radar images, DL is further used in estimations of rainfall and tropical cyclone intensity^37–39^. DL is also employed as a useful tool for forecasting air temperature, rainfall, and air quality^40–43^. Though based on a purely data-driven approach, DL can even simulate physical laws. Some studies tried to substitute DL for the physical models in weather forecasts, and DL performed much better than some simple physical models, but it has not beaten the operational, dynamical models yet^44–46^. Furthermore, an approach of physics-informed ML suggests guiding ML with known physical laws to improve the forecasts^47^. Meanwhile, the hybrid approach combining ML with physical modeling is the most applied one^25^. For example, DL is used for data assimilation and moist convection parameterization in climate models^48–50^. DL is applied for postprocessing of model outputs to remove systematic errors and allow the nonlinearity among ensemble members of the models^24,51,52^. Driven by the output data of the dynamical models, DL has yielded promising climate predictions for the East Asian Summer monsoon and El Niño Southern Oscillation^53–55^.

Minimum 2-m air temperature (T2Min) is one of the important variables for identifying extreme weathers in winter^56,57^. The cold events are usually defined based on the T2Min values^58^. Thus, the skillful prediction of T2Min is substantial for the prediction of extreme cold weathers. Changes in the position and strength of jet streams can significantly affect the atmospheric circulation and storm tracks over the mid-latitude Northern Hemisphere in winter, which can alter the weather patterns^59–63^. Therefore, the variations of the jet streams have significant influences on the winter air temperature over Asia^64–66^. Furthermore, jet streams are one of the major linkages between severe winters over the Northern Hemisphere and other climatic factors^67,68^. Recently, a study has also pointed out that jet stream and MJO have the joint influence on the S2S winter patterns over the Northern Hemisphere^69^. Generally, MJO is a useful signal with a long lead time of prediction in the model. Due to the quasi-periodic nature of MJO, the atmosphere can be influenced by MJO several weeks later, which indicates these kinds of signals can be used for prediction. The jet streams are important factors affecting winter temperature over Asia, and the present study is going to reveal that the concurrent variation of the subtropical and polar jet streams is another quasi-periodic signal over the midlatitude of Asia. On the other hand, DL is a useful tool for the postprocessing of model outputs, which can incorporate nonlinearity and even physical laws, though it is a black box that is difficult to track any physical process. Since MJO is well predicted by the model and MJO and jet streams have quasi-periodic nature, DL can be used to combine these kinds of signals and the atmospheric variables in postprocessing model outputs to improve the S2S prediction. In other words, we can use DL to transfer the benefit from the prediction of quasi-periodic signals to the prediction of the variables cared by the public. Based on this idea, a postprocessing approach of DL is proposed for combining the quasi-periodic signals and T2Min predicted by the operational S2S model of CMA (BCC model) to improve the S2S prediction of T2Min over Asia in boreal winter. This can potentially contribute to the S2S prediction of severe cold weathers over Asia.

## Results

### Quasi-periodic features of jet and MJO

**Figure 1:**
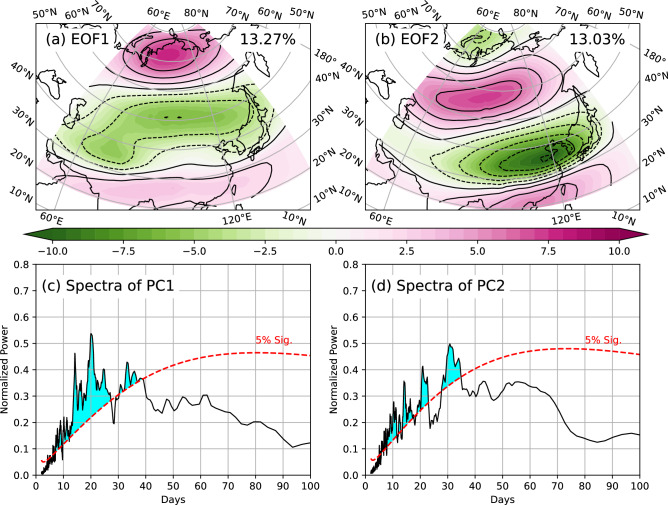
First two EOFs (panels **a** and **b**) of the daily 1.5° × 1.5° U_ERA_ at 200 hPa during boreal winter. Before the EOF, the seasonal cycle has been removed. The first EOF (EOF1) explains 13.27% of the total variance, and the second (EOF2) explains 13.03%. EOF is conducted over the region of 49.5–148.5°E and 19.5–79.5°N (shaded area). The two panels share the same color bar. Power spectra (black curve) for the two PCs of the first two EOFs (JET1&2) are shown in panels c and d, respectively. The red-dash line shows the 5% significant level based on the Markov red noise, and the significant power spectra are shaded in blue. The x-axis is the period, and the y-axis is the normalized power spectral.

In order to explore the quasi-periodic features of jet streams, EOF analysis as described in Methods is conducted on daily U_ERA_ at 200 hPa over Asia during winter (Fig. 1). The first EOF (EOF1) explains 13.27% of the total variances and shows a tripole pattern (Fig. 1a). A positively anomalous center of the U_ERA_ is over the polar region around 70°N. A negatively anomalous band is around 50°N (near the polar jet stream), and a weak positively anomalous band is around 25°N (around the subtropical jet stream). EOF1 generally reflects that when the zonal wind anomalies are strong and westerly wind increases over the polar region, the polar jet stream weakens and the subtropical jet stream enhances, and vice versa. Moreover, intensities of the polar and subtropical jet streams present opposite or seesaw variations. The second EOF (EOF2) explaining 13.03% of the total variances mainly presents a seesaw pattern over Asia (Fig. 1b), and two strong anomalous bands are around 55°N and 35°N with opposite signs. This pattern also presents the opposite variations of intensities between the polar and subtropical jet streams. Compared with EOF1 (Fig. 1a), the intensity of the zonal wind anomalies of EOF2 (Fig. 1b) is strong, and the locations of the seesaw anomalous bands are further north. Besides, the intensity of zonal wind over the polar region is weak, and there is a positively anomalous band around 20°N (Fig. 1b). The first two EOFs indicate a concurrent variation of both intensity and location of the two jet streams on S2S time scales over Asia during winter. To further explore features of this variation, spectral analysis is conducted on the time series of the two PCs (JET1&2) and shown in Figs. 1c and d. Significant spectra are found during 10–40 days for both JET1 and JET2. The significant spectral peaks are on 10, 15, 20, and 35 days for JET1 (Fig. 1c) and on 10, 15, 20, and 30 days for JET2 (Fig. 1b). Therefore, the concurrent variation of the two jet streams has significant oscillation on S2S time scales, and the bivariant indices of jet streams also present the propagation of those S2S signals. For the MJO, its convection mainly propagates eastwardly in the tropics, which was explored by many previous studies^4–6,70^.

**Figure 2:**
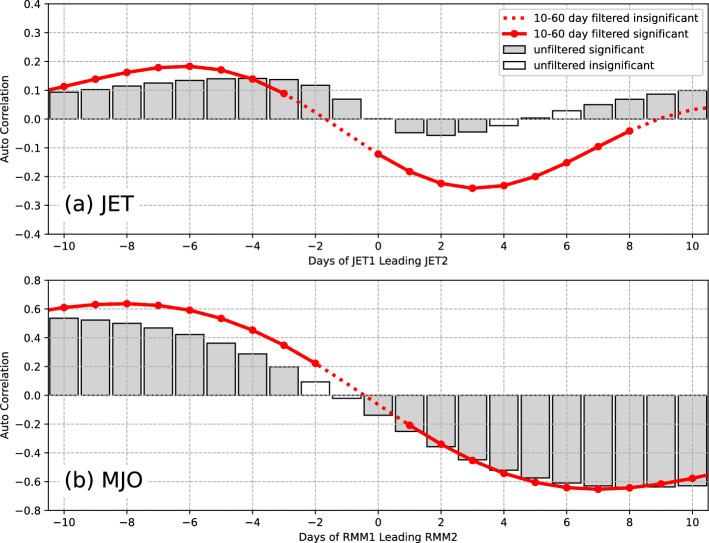
(**a**) Lead-lag correlation coefficients between JET1 and JET2 during the winters of 1979–2019, as well as those between RMM1 and RMM2 (**b**). The correlation coefficients for the unfiltered data are shown in bars, and those for 10–60-day filtered data are shown in red lines. The significant correlation coefficients at the 5% level are presented by filled bars or solid lines.

To further reveal the quasi-periodic nature of jet and MJO, Fig. 2 shows the lead-lag correlation coefficients between JET1 and JET2 during the winters of 1979–2019, as well as those for the MJO indices of RMM1 and RMM2. Due to those oscillations being on S2S time scales, band-pass filtering with the cutoff of 10–60 days using the Butterworth filter is also conducted on the time series of those indices. The correlation coefficients are generally weak and insignificant around day zero because of the orthogonal feature of the PCs of EOF analysis. For the jet stream (Fig. 2a), when JET1 leads JET2, the largest correlation coefficients are positive on days 4 and 6 for unfiltered and filtered data, respectively. The positive correlation indicates EOF1 leads EOF2, and vice versa. When JET2 leads JET1, the largest correlation coefficients are negative on days 2 and 3 for unfiltered and filtered data, respectively. For MJO (Fig. 2b), when RMM1 leads RMM2, the largest correlation coefficients are on days 10 and 8 for unfiltered and filtered data, respectively. When RMM2 leads RMM1, the largest correlation coefficients are on days 8 and 7 for unfiltered and filtered data, respectively. Except around day zero, both the unfiltered and filtered data generally exhibit significant lead-lag correlations between the two components of the bivariant indices of either MJO or jet. This indicates that the first two EOF patterns of either jet and MJO substitute each other on S2S time scales as time varying. Thus, each component of either indices of those two quasi-periodic signals incorporates future information of the other component for about 4–10 days. Overall, the two indices of MJO and jet have quasi-periodic features both spatially and temporally and their signals are time lagged, which can be applied for the usage of prediction.

The linear correlations between RMMs and JETs are insignificant, and there are significant but weak linear correlations between RMMs/JETs and T2Min, except JET2 (figures not shown), which is mainly because the significant influences of MJO and jet streams on air temperature over Asia^18,64–66^. In the following, RMMs and JETs are used as input features for DL models.

### Postprocessing by the DL models over Asia

**Figure 3:**
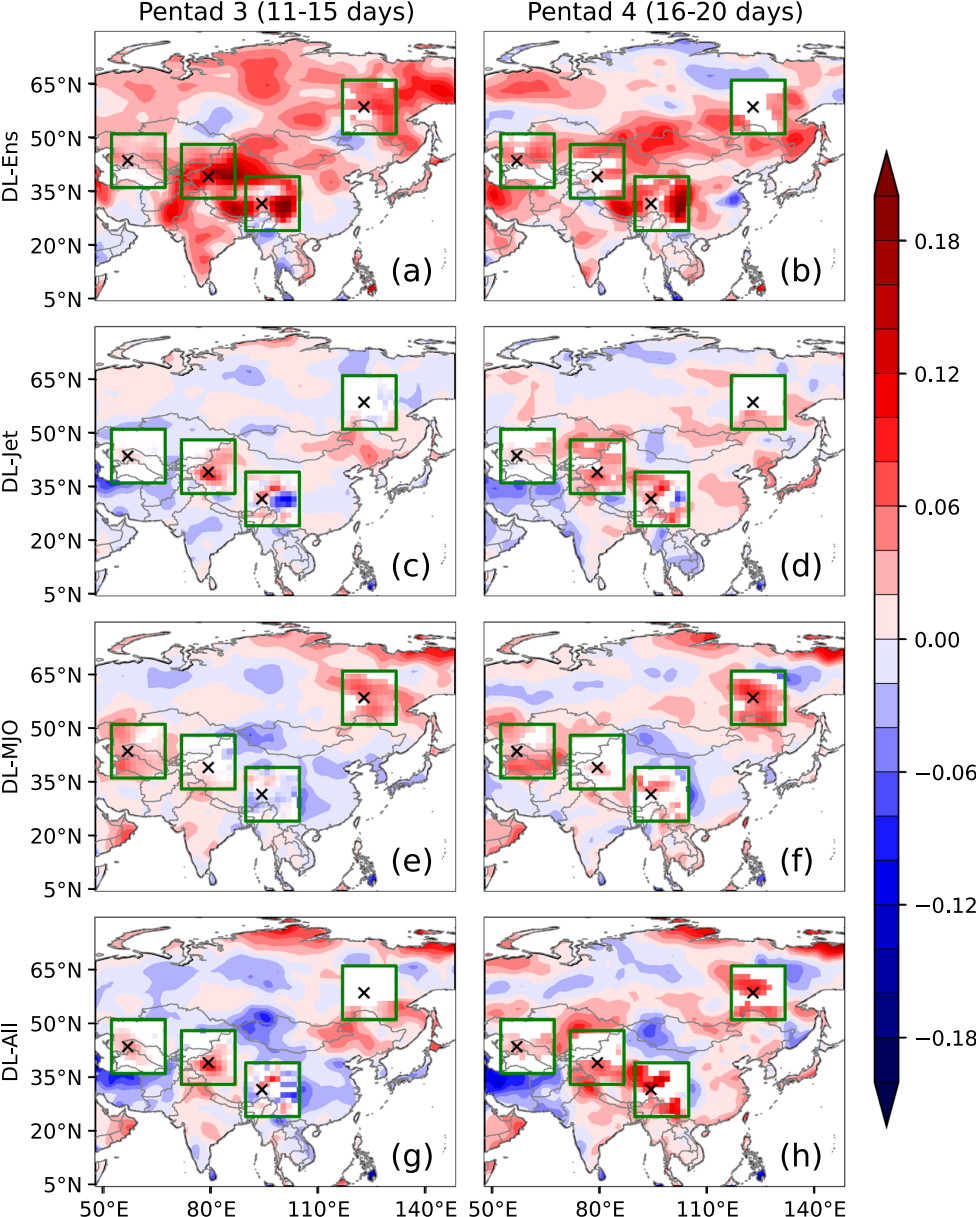
Differences of the correlation skill (R) among the direct mean of ensemble members and the four DL models at pentad 3 (left panels) and 4 (right panels). (**a** and **b**) Differences between R_DL-Ens_ and the direct mean of ensemble members. (**c** and **d**) Differences between R_DL-Jet_ and R_DL-Ens_. (**e** and **f**) Differences between R_DL-MJO_ and R_DL-Ens_. (**g** and **h**) Differences between R_DL-All_ and R_DL-Ens_. Due to the large consumption of the computation resources for the significant test, four regions surrounded by green rectangles are chosen for the significant test, and the grid cells filled with color are significant at the 5% level. Those regions are denoted as REG1, 2, 3, and 4 from left to right. The cross markers are used for Figs. 5 and 6. The DL models are built based on the pytorch module (version 1.10.0, https://pytorch.org/) of python. The figure is drawn using the modules of matplotlib (version 3.4.1, https://matplotlib.org/) and cartopy (version 0.18.0, https://scitools.org.uk/cartopy/) of python.

**Figure 4:**
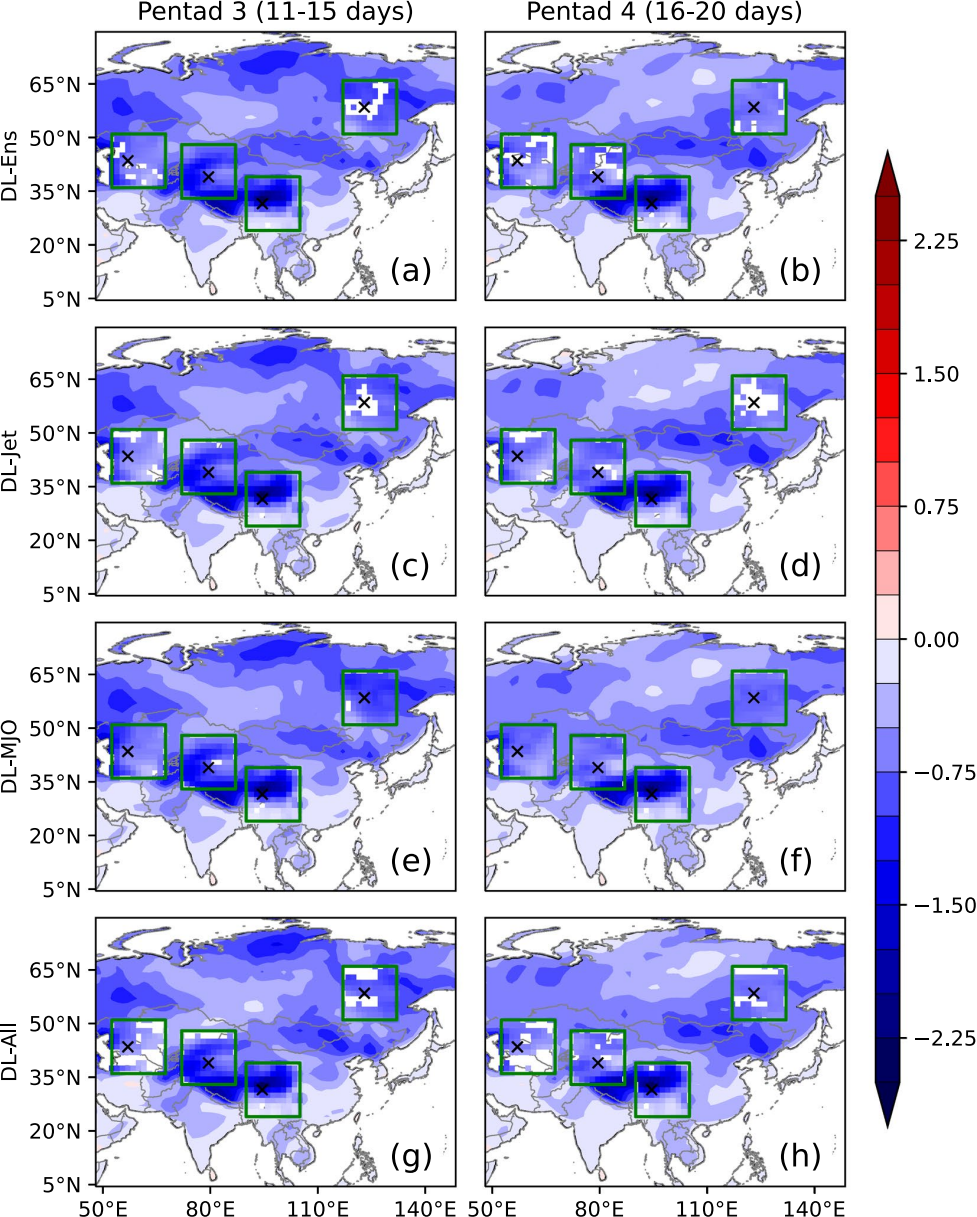
Same as Fig. 3, but for the differences of RMSE.

Using the T2Min, JET1, JET2, RMM1, and RMM2 from the S2S products of the BCC model, four DL models (Table 1 in “Methods” section) are trained against the T2Min of ERA5 during winters of 2004–2014 (10 winters). The four models have input features from 4 to 20 with a sequence length of 30 and batch size of 26 (30 × 26 × N, N indicates the size of input features). The output of a model is a matrix of 30 × 26 of T2Min, which are the sequence length and batch size, respectively. The sequence length is the length of forecast days, and batch size is the number of forecasts in a winter. Predictions of the DL models are validated during the winters of 2015–2019 (5 winters). The BCC model generally has skillful prediction during the first 10 days for T2Min, and thus the improvement of correlation skill (R) and RMSE for the four DL models over Asia at pentad 3 (11–15 forecast days) and 4 (16–20 forecast days) is shown in Figs. 3 and 4. The prediction of T2Min is averaged at pentad 3 and 4, and then R and RMSE are calculated against T2Min of ERA5.

**Table 1:** Four DL models with different numbers of features (N) for the input layer. For the BCC model, each of T2Min, RMM1, RMM2, JET1, and JET2 at a grid cell has the predictions of four ensemble members.

DL Model Name	Number of Input Features	Features
DL-Ens	N = 4	T2Min
DL-Jet	N = 4 + 2 × 4 = 12	T2Min and JET1&2
DL-MJO	N = 4 + 2 × 4 = 12	T2Min and RMM1&2
DL-All	N = 4 + 2 × 4 + 2 × 4 = 20	T2Min, JET1&2, and RMM1&2

Figures 3a and b show the differences in the skill between DL-Ens and the direct mean of ensemble members (∆R_DL-Ens_). Compared with the direct mean of the ensemble members, DL-Ens improves the prediction skill of T2Min over most parts of Asia. At pentad 3 (Fig. 3a), the improvement is found over most parts of Russia, southern Kazakhstan, Mongolia, western China, Pakistan, and India. At pentad 4 (Fig. 3b), areas with the improvement are similar to those at pentad 3, except northern Russia and part of central Asia. At both pentad 3 and 4, the largest improvement is over western China, and the prediction skill is generally worsened over northern Kazakhstan and southeastern China. In order to check whether jet and MJO can improve the prediction skills, the differences in the prediction skills between DL-jet and DL-Ens are presented in Figs. 3c and d, as well as those between DL-MJO/DL-All and DL-Ens (Figs. 3e-h). When the jet signal predicted by the BCC model is considered in the DL-Jet, compared to DL-Ens, the prediction is improved over western and northeastern China at pentad 3 (Fig. 3c) and eastern Kazakhstan, western and eastern China, and southeastern Russia at pentad 4 (Fig. 3d). When the MJO predicted by the BCC model is considered in the DL-MJO, compared to DL-Ens, the prediction skill is increased over central Asia, part of western China, and eastern Russia at both pentad 3 and 4 (Figs. 3e and f). When both the jet and MJO predicted by the BCC model are used in DL-All, prediction is improved over western and northeastern China at pentad 3 (Fig. 3g) and over Kazakhstan, western China, and eastern Russia at pentad 4 (Fig. 3h).

In the regions surrounded by green rectangles (Fig. 3), the significant test is conducted for the improvement of the prediction skill at each grid cell, and significant improvement is filled with color. For the color-filled grid cells over the four regions, the prediction skill is generally significantly improved (red) at pentad 3 and 4, except for some grid cells over central China, where the prediction skill is significantly decreased (blue). Contrary to those areas with improved prediction skills, some regions are with a decrease of the prediction skill postprocessed by the DL models. Thus, the applicability of DL method exhibits some regional dependency.

The same as Fig. 3, the RMSE at pentad 3 and 4 is shown in Fig. 4. It is noted that the seasonal mean and mean of both model results and observations are removed before the calculation of RMSE, and thus RMSE is unbiased. The RMSE is decreased in DL-Ens compared with the direct mean of ensemble members. When the jet and MJO predicted by the BCC model are considered, the RMSE at pentad 3 and 4 in DL-Jet, DL-MJO, and DL-All is also decreased compared with DL-Ens. In Fig. 4, RMSE is either decreased or unchanged but not increased, and the largest decrease of RMSE is over the Tibetan Plateau. In the four regions surrounded by the green lines, most parts are with a significant decrease in RMSE. Therefore, the DL method can significantly decrease the error of the BCC model, though it cannot increase the prediction skill over entire Asia.

### Time variations of prediction skill of the DL models

**Figure 5:**
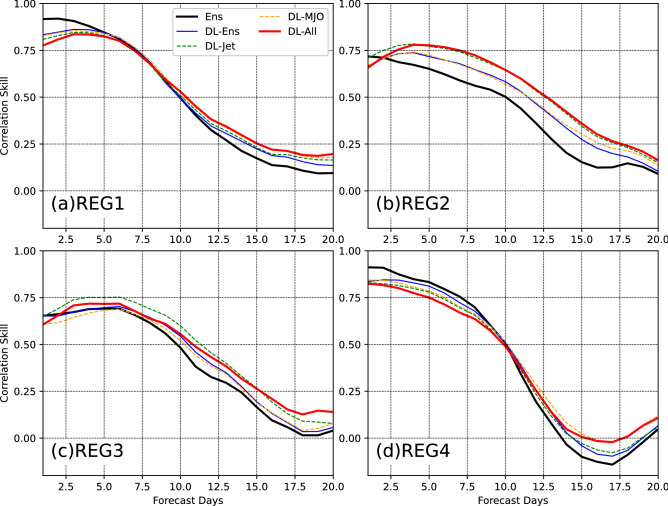
Time variations of R at four grid cells in the four regions marked in Figs. 3 and 4. The R values are presented during the first 20 forecast days. The solid black curve is the R for the direct mean of ensemble members. The solid blue, dash green, dash orange, and solid red lines are for DL-Ens, DL-Jet, DL-MJO, and DL-ALL, respectively. The y-axis and x-axis are for R and forecast days, respectively.

**Figure 6:**
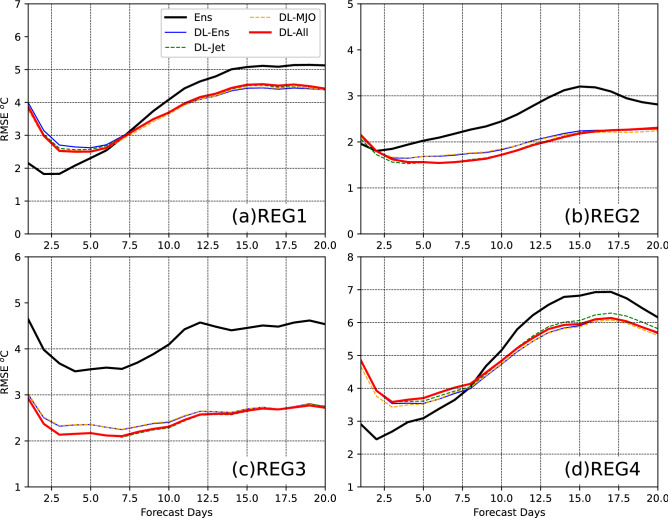
Same as Fig. 5, but for RMSE.

To further compare the DL models and present time variations of the prediction skill, the time variations of R and RMSE during the first 20 forecast days are presented in Figs. 5 and 6. The R and RMSE are calculated at the four grid cells in the four regions shown by the cross markers in Figs. 3 and 4.

For the BCC model, the direct mean of the ensemble members has the prediction skill greater than 0.5 during the first 10 forecast days at the four points (Fig. 5). R ≥ 0.5 indicates the skillful prediction of T2Min. During the first 10 forecast days, the prediction skill in REG2 and 3, where are western China and the eastern Tibetan Plateau, are smaller than that in REG1 and 4, where are central Asia and eastern Russia. After 10 days, the prediction skill is not skillful at all four points, and thus the BCC model generally has the ability to the prediction T2Min with a lead time of 10 days. After postprocessing by the DL models, the prediction skill has been improved after 10 forecast days. During the first 10 forecast days, the skill is decreased at the points in REG1 and 4 but increased in REG2 and 3 in DL-models.

Because the prediction during the first 10 forecast days is skillful in the BCC model, we mainly focus on the improvement of the prediction after 10 forecast days. In REG1 (Fig. 5a), the prediction skill is improved by all the DL models after 10 forecast days, and the improvement of DL-All is the largest among them with the improvement of lead days of about 0.5. DL-Jet and DL-Ens almost have the same improvement, and DL-MJO and DL-All almost overlap with each other. This indicates that MJO contributes more to the improvement by the postprocessing at the point in REG1. In REG2 (Fig. 5b), DL-All also has the largest improvement among the forecasts with the improvement of lead days up to 2.5 days. DL-MJO and DL-Ens have the similar improvement, and DL-All and DL-Jet almost overlap with each other. Thus, jet has larger contribution to the postprocessing than MJO at the point in REG2. In REG3 (Fig. 5c), the prediction of DL-Jet has the largest skill during 10–15 days among the forecasts, but that of DL-All has the largest during 15–20 days. The jet contributes more to the prediction skill than MJO at the point in REG3. In REG4 (Fig. 5d), DL-Ens, DL-Jet, and DL-All almost have the same improvement of the skill during 10–15 days. During 15–20 days, DL-All and DL-MJO have the largest improvement. At this point in REG4, MJO contributes more to the prediction than the jet. Similar results can be found at most of the points in the four regions, which can be also derived from Fig. 3. When considering MJO predicted by the BCC model in the DL postprocessing, the prediction skill is improved over central Asia and eastern Russia. When considering jet predicted by the BCC model in the DL postprocessing the skill is improved over western China and the eastern Tibetan Plateau. This further proved that the applicability of DL method exhibits some regional dependency. For the RMSE (Fig. 6), the four DL models present negligible differences and similar variations during the 20 forecast days. At the points in REG1 and 3, the RMSE is increased by the DL models before 7.5 forecast days but decreased after then. At the points in REG2 and 3, the RMSE is generally decreased during 20 forecast days.

Overall, the DL postprocessing can generally increase the prediction skill and decrease the RMSE compared to the direct mean of ensemble members over Asia. Quasi-periodic signals (e.g., jet and MJO) cannot improve the prediction everywhere over Asia but can improve the skill over some specific regions.

### Discussions

**Figure 7:**
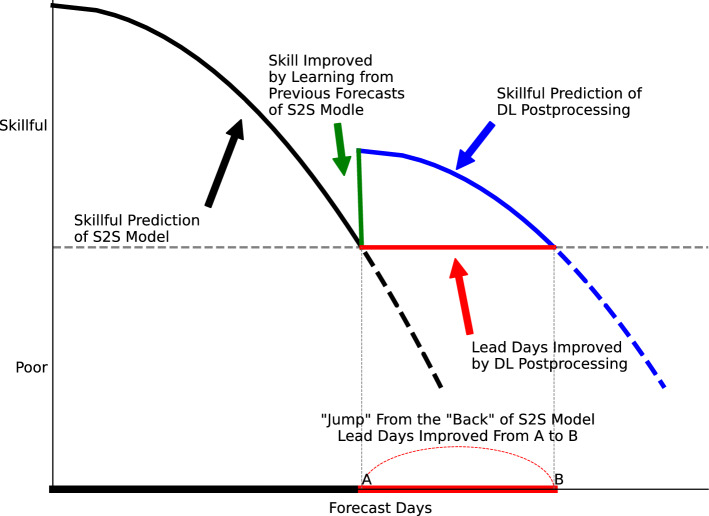
Schematic diagram for the DL approach improving the prediction skill of S2S operational models.

The T2Min in the BCC model generally has skillful prediction during the first 10 forecast days, after then the skill drops. In the present study, DL can find the best weights for the ensemble members to significantly improve the prediction during postprocessing. Like many DL postprocessing, this approach only uses the predictions of T2Min itself, because the nonlinearity and sequence dependence are considered by the LSTM model. Besides the prediction of T2Min itself, there are many other factors predicted by the model, which have better prediction skills or quasi-periodic nature. Moreover, those factors can influence the T2Min psychically in the observation, and many previous studies have illustrated that MJO and jet stream have significant effects on the midlatitude climate^4–6,9,17,18,71,72^. MJO and jet have quasi-periodic features, and the model can predict them well in the first 10 forecast days. Thus, using the DL approach, the information of MJO and jet that are well predicted in the previous time can be used to correct the prediction after then according to the quasi-periodic nature of the signals (Fig. 7). Moreover, the prediction skill of MJO is higher than that of T2Min in the BCC model, and those skills can be also used by the DL approach to improve the prediction of T2Min, though it is difficult to separate this effect from other effects in the DL models. The DL approach in this study suggests using the information that is well predicted in the first two weeks of the forecast to improve the forecast after then, based on the quasi-periodic feature of that information (Fig. 7). It is like an approach to “jump” from the “back” of S2S model to improve the lead days of the prediction of T2Min. Thus, with the improvement of the numerical model, the DL postprocessing could always have a better prediction than the numerical model itself.

It is interesting to find that the prediction skill at pentad 4 is improved more than that at pentad 3 in the DL models. We think this probably because the useful information can be provided by the LSTM model is already provided by the numerical model at pentad 3. Thus, less useful signal is provided by the LSTM model for improvement. At pentad 4, the numerical model losses more information from the initial condition than at pentad 3. When the prediction skill in the numerical model drops faster than the memory of the LSTM model, the LSTM model can provide more useful information than that at pentad 3. Therefore, the prediction skill is improved more at pentad 3 than at pentad 4.

It is also noted that the LSTM network does not characterize the spatial motion of the atmosphere. LSTM is chosen because we mainly want to get the prediction at each grid (or location) that is only the result of the signals in jet/MJO but not the atmospheric variables on other grids. Although the spatial motion of the atmosphere over the grid is the reason for the effects of jet/MJO on prediction, it is not considered in the present study. On the other hand, the dynamic model already includes the results of the spatial motion of the atmosphere. This part maybe not well captured by the dynamic model, and a DL model has the potential to improve it. However, it is another interesting issue that can be further explored.

The DL-Ens is an approach to find the best weights for the ensemble members for the S2S products, and prediction skill is significantly improved over most parts of Asia. This indicates that the direct mean of the ensemble members (equal weights) can be improved, though its prediction skill is better than any single member. DL-Jet, DL-MJO, and DL-All consider the signals of jet and MJO during the postprocessing, and the prediction skill is further improved against DL-Ens. This indicates that those signals interact with T2Min and can provide useful information for correcting its prediction. However, the prediction skill is not improved all over Asia, and prediction skill is worsened in some regions. This indicates that the signals used for postprocessing have regional dependency, whereas the usage of the right signal can improve the prediction for a specific region and the wrong signal may worsen the prediction in the DL models. It is interesting to see that the regions with significant improvement are significantly affected by jet and MJO documented by previous studies. For example, the 2-m air temperature over central Asia is significantly affected by MJO^18^. Western China and the eastern Tibetan Plateau are significantly influenced by the subtropical jet stream^73^. So far, less study has explored the influence of MJO on the temperature variation over eastern Russia. Except for jet and MJO, there are many other quasi-periodic signals in the atmosphere, land, and ocean, which can have significant effects on specific regions and variables. Therefore, specific DL models can be designed for specific regions with the quasi-periodic signals that affect the climate in those regions for any operational model. Although there are still some unsolved issues, it is generally clear that jet and MJO have significant influence on the air temperature over those regions, except for REG4. The present study didn’t focus on a specific physical phenomenon, and thus it is difficulty to provide specific physical explanations. Though the improvement of the prediction skill seems to be specific in the DL model, the mechanism can be very complicated, which can not be explained by a single work. On the other hand, for the DL model, the mechanism behind is a worldwide challenge for DL applied in physical world. As the technology improved fastly, the DL structure will be changed, and the mechanism for an old DL structure may not suitable for a new one.

## Conclusions

The quasi-periodic signals in the earth system are “treasures” for S2S climate prediction. The quasi-periodic features of the jet streams are explored from the daily zonal wind at 200 hPa of ERA5. Significant periodic power is identified in 10–40 days for the concurrent variation of the subtropical and polar jet streams over Asia, which includes the concurrent variation of both intensity and location. Compared to the jet streams, MJO is the dominant S2S variability in the tropics and also has quasi-periodic nature.

Like many previous studies, the results of DL-Ens show that the DL approach can find the best weights for the ensemble members to significantly improve the prediction skill than the direct mean of ensemble members. Furthermore, using reforecasts of the S2S forecast from the BCC operational model of CMA, the DL models are constructed applying quasi-periodic signals predicted by the BCC model to postprocess the T2Min prediction during boreal winter over Asia. The DL models include LSTM layers that considered the sequence dependencies in the time series, and thus the quasi-periodic signal can be passed to the future period to improve the prediction of T2Min during 11–20 days. Moreover, the advantage of the well-predicted MJO can be privileged for the improvement of the prediction of T2Min. When the jet is considered in the DL models, the prediction skill over western China and the eastern Tibetan Plateau is significantly improved compared to DL-Ens. When MJO is considered in the DL models, the prediction skill over central Asia and eastern Russia is significantly improved compared to DL-Ens. The RMSE is generally decreased by the DL models over entire Asia. This study suggests the further application of the DL approach in the postprocessing of S2S forecasts considering the usage of quasi-periodic signals as input features. Due to the regional dependency of the DL models, especially its input features, the method presented in this study can be further applied in specific regions with specific input features for the improvement of S2S prediction of an operational model or multi-models.

So far, the S2S prediction of severe cold weathers in winter is still a challenge for operational centers. Applying DL methods during the postprocessing of dynamical model outputs, to a certain extent, can improve the T2Min prediction. In other words, there is a great potential for the application of DL methods to predict extreme cold weathers. However, there are still unsolved issues for future studies. For example, while the T2Min prediction is improved, can we capture the extreme cold events? Moreover, this study only considered outputs of a single dynamical model, but the DL method is a data-driven approach. If outputs of multi-models are used, can we further improve the prediction when the quasi-periodic signals are considered? Are there other quasi-periodic signals that can benefit S2S prediction? Finally, the present study proves that quasi-periodic signals in the atmosphere do benefit the S2S operational prediction with the help of deep learning.

## Methods

### Data

The hourly 2-m air temperature of ERA5 reanalysis^74^ with the horizontal resolution of 0.25° × 0.25° during 2004–2019 is provided by the European Center for Medium-Range Weather Forecasts (ECMWF). The daily T2Min is obtained through identifying the minimum value of the hourly 2-m air temperature during 24 h. The hourly zonal wind (U_ERA_) at 200 hPa of ERA5 with a horizontal resolution of 0.25° × 0.25° during 1979–2019 is averaged into daily data for analysis. Both T2Min and U_ERA_ of 0.25° × 0.25° are binned averaged into the horizontal resolution of 1.5° × 1.5°, which is for the comparison with the S2S products of the BCC model. The ERA5 data can be downloaded on the website of https://cds.climate.copernicus.eu/.

Besides the ERA5 reanalysis, the daily zonal winds of the US National Center for Atmospheric Research/Department of Energy reanalysis 2 (NCEP)^75^ at 850 and 200 hPa during 1979–2019 are with a horizontal resolution of 2.5° × 2.5°. The daily interpolated outgoing longwave radiation (OLR) of the US National Oceanic and Atmospheric Administration during 1979–2019 has a horizontal resolution of 2.5° × 2.5°. Both the NCEP zonal winds (U_NCEP2_) and OLR data are obtained on the website of https://psl.noaa.gov/data/gridded/index.html.

The S2S reforecasts of the BCC model during 2004–2019, including T2Min, OLR, and zonal winds at 850 and 200 hPa, are provided by CMA. The BCC model is a fully-coupled climate system for S2S operational prediction, which is running with fixed initial dates (twice a week) in a year. Each running is integrated for 60 days with four ensemble members, which are initialized at 18, 12, and 06 UTC of the day before the first forecast day and 00 UTC of the first forecast day. The atmospheric module of the BCC model has a horizontal resolution of approximately 45 × 45 km (T266) and 56 sigma-pressure hybrid vertical levels. The outputs of the reforecast are interpolated into the horizontal resolution of 1.5° × 1.5° and pressure levels to satisfy the requirements of the S2S project (http://www.s2sprediction.net). Details about the BCC model and its S2S outputs can be referred to^76^. The first-30-day reforecasts initialized during boreal winter (December-February) are analyzed in the present study. It is noted that on the website of the S2S project, the BCC model provides the reforecast only during 2004–2018.

### MJO index

The index of MJO proposed by^70^ (WH04) for monitoring MJO is provided by the Bureau of Meteorology of Australia (http://www.bom.gov.au/climate/mjo/). The WH04 index is bivariate that includes two time series (RMM1&2), which are the first two principle components (PCs) of the Empirical Orthogonal Function (EOF) of OLR and zonal winds at 850 and 200 hPa of NCEP reanalysis 1 between 15°S and 15°E. Before conducting the EOF analysis, the seasonal cycles and mean are removed from all the data. For real-time monitoring, the first two EOFs derived from the data during 1979–2001 are saved, and then the real-time OLR and zonal winds are projected onto the EOFs to obtain the bivariant MJO indices.

Following the same recipe, the OLR and U_NCEP2_ at 850 and 200 hPa are used to obtain the first two EOFs during 1979–2001 in this study. After that, the data during 1979–2019 is projected on the two EOFs to obtain the observed MJO indices. The correlation coefficients between the obtained indices and those directly download from the website are 0.94 and 0.97 for RMM1 and RMM2, respectively, and the differences are negligible. For the S2S products of the BCC model, the prediction is firstly bin averaged into the horizontal resolution of 2.5° × 2.5°and then projected onto the first two EOFs mentioned above to obtain predicted MJO indices for the four ensemble members. The bivariant correlation of MJO indices between the ensemble prediction and observations is greater than 0.5 during the first 23 forecast days in boreal winter. This indicates that the lead time of MJO prediction with useful skill is about 23 days in the BCC model during boreal winter, which is consistent with the results of previous studies^20,21^.

### Jet index

In addition to MJO, the jet streams over Asia, including subtropical and polar jet streams^77^, also have quasi-periodic nature on S2S time scales. In order to present their quasi-periodic feature on S2S time scales, indices are needed to represent their activities over Asia on S2S time scales. Referring to the idea of the MJO index of WH04, EOF analysis is conducted on the daily U_ERA_ with the resolution of 1.5° × 1.5° at 200 hPa over the region of 49.5–148.5°E and 19.5–79.5°N, which is the major region for the jet stream activities over Asia. The seasonal cycle is firstly removed from the data, and then the first two EOFs of 200-hPa U_ERA_ during the winters of 1979–2008 are obtained. After that, the data during 1979–2019 is projected on the first two EOFs to obtain bivariant indices (JET1&2) representing the activities of jet streams over Asia. For the S2S product of the BCC model, the zonal wind at 200 hPa is projected onto the first two EOFs mentioned above to obtain the prediction of JET1&2 during the winters of 2004–2019 for the four ensemble members. The bivariant correlation coefficient is calculated for JET1&2 between the model and ERA5, and the useful prediction skill for the bivariate indices of the jet has a lead time of about 10 days in the BCC model.

### Deep learning

**Figure 8:**
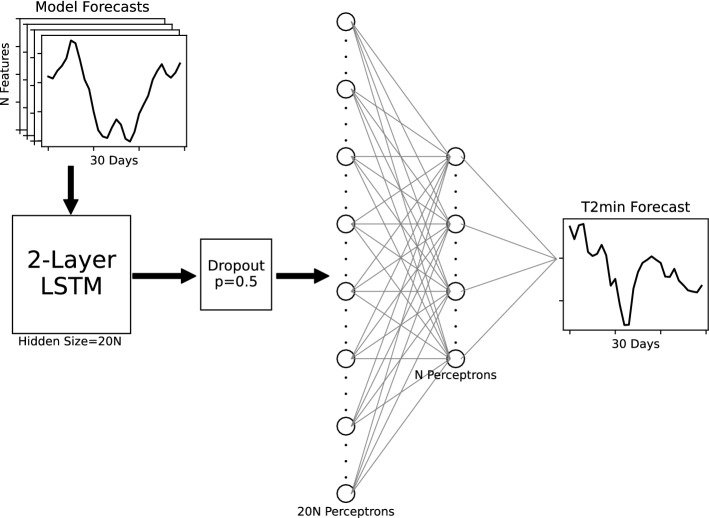
Structure of the DL models. It has an input layer (top left) with 30-day forecasts from the BCC model as input features with the number of N, including ensemble members of the prediction for T2Min, JET1&2, and MJO1&2. Following the arrows, the N features are passed to two LSTM layers with a hidden size of 20N and then a dropout layer that randomly sets elements to zero to avoid overfitting with the probability of 0.5. After that, the 20N features are passed to two linear (or multi-perceptron) layers with 20N and N perceptrons (circles), respectively. The value of N is presented in Table 1. Finally, the output layer (middle right) is the postprocessed 30-day prediction of T2Min. The activation function of hyperbolic tangent (Tanh) is used in between two layers to incorporate nonlinearity.

Using the first-30-day forecasts of T2Min, RMM1&2, and JET1&2 at a grid cell in the BCC model as input features of a DL model (Fig. 8), the prediction of T2Min is postprocessed against the T2Min of ERA5. In Fig. 8, the DL model has an input layer with the BCC model forecasts as input features (number of N features). The features are inputs of two long short-term memory (LSTM) layers with 20N as the size of the hidden features. LSTM is recurrent neural networks designed for remembering signals for a long time period, which means it is capable of learning long-term dependencies^78^. Thus, LSTM is suitable for processing speech recognition and the time series with dependencies^79^, for example quasi-periodic signals. In the present study, the LSTM models have two hidden layers that are bidirectional. To avoid overfitting the LSTM networks, a dropout layer with a dropout rate of 0.5 is set after the LSTM layers^80^. Following the dropout layer (Fig. 8), there are two linear (or multi-perceptron) layers with 20N and N perceptrons, respectively. At last, the output layer is the postprocessed T2Min of the 30-day forecast for the BCC model. In the DL model, the activation function of hyperbolic tangent (Tanh) is used to connect two layers to incorporate nonlinearity. In order to enlarge the sample size for training, the 260 forecasts of 10 winters are randomly shuffled 10 times. After each shuffle, the 260 forecasts are resized into the size of 26 × 10, and 26 is used as the batch size. After shuffled 10 times, the forecasts have the size of 26 × 100, which have a batch size of 26 and a sample size of 100. Due to the enlargement of the sample size, the epoch used in the model is 10, which yields results that are no different from 50, 100, or 200 epochs (tested at several grid cells). Thus, the small size of the epoch is used to save the computation time. Moreover, the optimization algorithm is the implement RMSprop algorithm with a learning rate of 1e-3, and the learning rate of each parameter group is decaying after every 2 epochs with a decay rate of 0.1. So far, from our trial and error, it is found that either introducing additional hidden layers of LSTM or enlarging the number of perceptrons in linear layers does not improve the predictions as additional DL-model complexity may increase the potential of overfitting.

Using the same structure (Fig. 8), four DL models with different input features are trained and used to postprocess 30-day prediction of T2Min (Table 1). For the S2S products of the BCC model, the predictions of four ensemble members are provided. In Table 1, the model DL-Ens uses the prediction of T2Min from the four ensemble members as the input features. This is synonymous with finding the best weights for the members to obtain the ensemble mean. DL-Jet and DL-MJO have the same input features of 12. Each model includes the predictions of both the T2Min and one kind of the bivariant indices from the four ensemble members, and thus effects of each kind of the indices on the results of postprocessing can be considered. The model DL-All uses T2Min, JET1&2, and RMM1&2 from the four ensemble members as input features, which is of the number 20.

During the winters of 2004–2019, the forecasts initialed in January and February of 2004 and December of 2019 are discarded. Therefore, there are 15 winters with 390 forecasts for analysis. Instead of randomly choosing the samples for training and to mimic operational conditions as closely as possible, the model forecast of the first 10 winters with the number of 260 forecasts is used for training the DL models to fit ERA5 T2Min, and the last five winters with the number of 130 forecasts are used for the DL model validation. Generally, 260 forecasts are used as training samples, and each forecast has 30 days of prediction, which means the sequence length for LSTM is 30. Furthermore, the 130 forecasts are used as testing samples to validate the performance of the DL models. The correlation coefficient (R) and root mean square error (RMSE) between the T2Min from the DL models and ERA5 are calculated for validation.

### Significant test

During the validation for the DL forecast of the last five winters, the significance of the improvement is investigated. In order to check whether the result of DL-Ens is significantly better than the direct mean of the four ensemble members on a forecast day or during a forecast period, the following steps are conducted. (1) The R and RMSE of DL-Ens are calculated (denoted as R_DL-Ens_ and RMSE_DL-Ens_, respectively) for the five winters, as well as those for the direct mean of the four ensemble members (denoted as R_Ens_ and RMSE_Ens_, respectively). The differences are calculated between R_DL-Ens_ and R_Ens_ (∆R_DL-Ens_), as well as those between RMSE_DL-Ens_ and RMSE_Ens_ (∆RMSE_DL-Ens_). (2) Due to the DL-Ens is equal to seek the best weights for the four ensemble members, four float values (seven decimal places) are randomly chosen between 0.0 and 1.0 and divided by their sum as the weights for the four ensemble members. This procedure is conducted 1000 times to obtain 1000 predictions through summing the four ensemble members with random weights. (3) The R and RMSE of the 1000 predictions are calculated, denoted as R_*i*_ and RMSE_*i*_ (1 ≤ *i* ≤ 1000). Meanwhile, the differences are calculated between R_*i*_ and R_Ens_ (∆R_*i*_), as well as those between RMSE_*i*_ and RMSE_Ens_ (∆RMSE_*i*_). (4) The percentages of the ∆R_*i*_ ≥ ∆R_DL-Ens_ > 0 and ∆RMSE_*i*_ ≤ ∆RMSE_DL-Ens_ < 0 are identified, denotated as *p*_*r*_ and *p*_*rmse*_, respectively. If the *p* value is smaller than 5%, it means that DL-Ens does not improve the forecast by chance, which indicates DL-Ens significantly improves the forecast at the 5% level. On the contrary, the percentages of the ∆R_*i*_ ≤ ∆R_DL-Ens_ < 0 and ∆RMSE_*i*_ ≥ ∆RMSE_DL-Ens_ > 0 can be also identified. If the *p* value is smaller than 5%, it means that DL-Ens does not worsen the forecast by chance, which indicates DL-Ens significantly worsen the forecast at the 5% level.

For the DL-Jet, DL-MJO, and DL-All, whether including MJO or jet signals can significantly improve the prediction of DL-Ens is examined. The procedure is similar to that for the significant test of DL-Ens, except for some differences. The time sequences of the indices of MJO and jet are randomly shuffled1000 times, and then the DL models are trained by those shuffled time sequences for obtaining R_*i*_ and RMSE_*i*_, and ∆R_*i*_ and ∆RMSE_*i*_ are calculated against R_DL-Ens_ and RMSE_DL-Ens_, respectively. After that, the *p* values for the significant test are obtained. During this procedure, we can ensure that whether those indices can significantly improve the prediction. Due to this procedure trains the model 1000 times at each grid cell, large consumption of computation resources is needed.

## Data Availability

All the data used can be downloaded freely from the website. ERA5 reanalysis is on the website of https://cds.climate.copernicus.eu/. NCEP reanalysis and OLR are downloaded on the website of https://psl.noaa.gov/data/gridded/index.html. S2S products of the BCC model can be found on the website of http://www.s2sprediction.net. The WH04 MJO index is on http://www.bom.gov.au/climate/mjo/.
